# Progress on the Mechanism of Visceral Hypersensitivity in Nonerosive Reflux Disease

**DOI:** 10.1155/2022/4785077

**Published:** 2022-01-20

**Authors:** Cao Xu, Xiaoping Niu

**Affiliations:** Department of Gastroenterology, Yijishan Hospital, Wannan Medical College, Wuhu, China

## Abstract

Nonerosive reflux disease (NERD) is the most common type of gastroesophageal reflux disease (GERD). Its clinical symptoms can recur, and clinical treatment is often ineffective, causing patients severe economic and psychological burden. In recent years, studies that have explored in-depth the pathogenesis of NERD have found that visceral hypersensitivity (VH) plays an important role. VH refers to the phenomenon that viscera react strongly to nociceptive stimuli or produce a negative reaction to physiological stimuli due to the decrease of one's visceral pain threshold. Studies have found that the VH mechanism in NERD primarily includes abnormal neurotransmitters, the activation of acid-sensitive receptors, and abnormal psychological factors—all of which we review in this article.

## 1. Introduction

GERD, a common digestive system disease, has an incidence rate of 8.8%–25.9% in Europe, 18.1%–27.8% in North America, 23% in South America, and 8.7%–33.1% in the Middle East [1]. In China, the overall prevalence of GERD is only 2.5%–7.8%, but it is increasing each year [2]. According to endoscopic findings, GERD can be divided into three subtypes: NERD, reflux esophagitis (RE), and Barrett's esophagus (BE) [3, 4]. NERD is the most common of the three, accounting for approximately 70% of GERD cases [5], and is mainly characterized by typical gastroesophageal reflux symptoms. NERD is also without mucosal injury under endoscope and thus is called an “endoscopic negative gastroesophageal reflux disease,” of which acid reflux and heartburn are typical symptoms [6]. Compared to those with RE, some NERD patients have poor response to proton pump inhibitors (PPIs), and their clinical symptoms often recur repeatedly. For these patients, long-term medical treatment and repeated examination not only require a significant expenditure of social resources but also severely affect patients' quality of life [7]. Because of the poor effectiveness of conventional treatment and NERD's recurrent symptoms, more attention has recently been paid to NERD. An increasing number of researchers have come to believe that VH plays an important role in the pathogenesis of NERD.

## 2. Abnormal Neurotransmitters and NERD

Substance P (SP) and calcitonin gene-related peptide (CGRP) are the most important neurotransmitters in pain signal transduction, and they play a key role in mediating the hyperalgesia caused by nociceptive or nonnociceptive stimulation. SP and CGRP coexist in the primary afferent neurons of spinal dorsal-root neurons, and overexpression can change the membrane potential of class C nerve fibers, thereby increasing their sensitivity. The high excitability of the spinal cord persists during this process, making the tissue abnormally sensitive to harmless or weak stimuli [8]. This overexpression of SP and CGRP may also lead to neurogenic inflammation, changes in the local microenvironment, and hyperalgesia [9]. Studies have found that SP and CGRP are involved in the pathogenesis of and play an important role in VH in NERD [10, 11].

### 2.1. SP and NERD

SP is mainly expressed in the esophageal submucosal nerve plexus and can be activated and released by transient receptor potential vanilloid 1 (TRPV1) and protease-activated receptor-2 (PAR-2) in the esophageal mucosa. When this occurs, the sensitivity of nerve fiber endings increases after repeated stimulation. SP not only acts as a pain transmitter to the central nervous system but also increases esophageal vascular permeability, causes local-tissue mucous edema, further increases the sensitivity of nociceptive receptors, and leads to the release of inflammatory mediators, growth factors, cytokines, and other related factors [12]. Similarly, the dorsal horn of the spinal cord can also release SP to sensitize the center of the dorsal horn of the spinal cord, thereby reducing the pain threshold. Yoshida et al. [13] reported that the expression of SP was upregulated in the colon of mice with VH, and the content of SP in the colonic mucosa of patients with irritable bowel syndrome (IBS) was also significantly increased. Similarly, a study conducted by Wang et al. [14] found that the levels of SP protein and its receptor NK1R mRNA in the esophageal mucosa of patients with NERD were significantly heightened (*P* < 0.05). These elevated levels of the SP protein were positively correlated with the reflux symptoms of NERD.

### 2.2. CGRP and NERD

CGRP is widely distributed in the central and peripheral nervous systems, including a high concentration in the dorsal horn of the spinal cord and the dorsal-root ganglion. CGRP is also highly expressed in the digestive system. The CGRP protein can inhibit endopeptidase related to SP degradation and increase or prolong the effect of SP, which has a synergistic effect in the process of mediating VH. Both can activate mast cells, causing them to degranulate and release inflammatory mediators and cytokines [9]. In addition, when the membrane potential of class C nerve fibers changes, the fiber endings can release CGRP to positively regulate the degranulation release of mast cells to maintain the high excitability of class C nerve fibers and induce VH. Deng et al. [15] compared model rats that exhibited IBS with normal rats and found that the expression of the CGRP protein in the colons of the rats with IBS was significantly higher than that among the control group (*P* < 0.01). Similarly, Xu et al. [8] found that the expression of CGRP in the esophageal mucosal epithelium of patients with NERD was significantly higher than that of normal patients and negatively correlated with patients' pain thresholds.

## 3. NERD and the Activation of Acid-Sensitive Receptors

The primary acid-sensitive receptors are TRPV1, acid-sensitive ion channels (ASICs), and PAR2. When esophageal sensory nerve endings are stimulated by chemical, mechanical, temperature, or pressure changes, the expression of these acid-sensitive receptors also changes accordingly and further affects the release of neurotransmitters or changes in the electrophysiology of the esophageal mucosa. This results in potential inflammation, immunity, hyperalgesia, and NERD VH (Table 1) [13, 16–21].

### 3.1. Transient Receptor Potential Vanilloid 1 (TRPV1)

TRPV1 is a nonselective ligand-gated cation-channel receptor and is widely distributed in viscera and sensory neurons. Hyperalgesia caused by digestive tract inflammation can be largely attributed to the upregulation and activation of TRPV1 expression [22, 23]. A study has shown that the expression of TRPV1 in the esophageal mucosa of patients with NERD is significantly higher than that in patients with erosive reflux disease (ERD) [20]. When the esophagus is injured or exposed to stress, local inflammatory activities such as vascular dilatation and plasma protein exudation will occur. These inflammatory responses can further activate TRPV1, reduce the signal transduction threshold of TRPV1, and thus increase the sensitivity of esophageal viscera. Activated TRPV1 can also induce neurogenic inflammation by promoting the release of neuropeptides, which also play a role in mediating VH.

### 3.2. Acid-Sensitive Ion Channels (ASICs)

ASICs are a class of non-voltage-sensitive cation channels activated by the extracellular proton H+. There are six subtypes in the ASIC family, among which ASIC1 and ASIC3 play an important role in the occurrence of gastrointestinal acid sensitivity [24]. ASIC3 is highly expressed in esophageal sensors, mainly located in peripheral nociceptors [25]. When the esophagus is stimulated by acid, the ASIC3 activated by H+ can produce a biphasic current of rapid inactivation and steady inactivation, resulting in electrophysiological disorder of the esophageal mucosa and a pain sensation caused by the disordered electrophysiological stimulation of sensory nerve endings. Observation under a microscope of the esophageal mucosa of patients with NERD revealed dilated intracellular spaces (DIS) [26], which was considered to be caused by reflux such as gastric acid, pepsin, and bile. The presence of DIS gradually increases the level of H+ entering and remaining in the submucosal intercellular space of the esophagus; the DIS also stimulates acid-sensitive nociceptive receptors and causes weak acid reflux and typical symptoms of heartburn and acid reflux [27]. At the same time, ASIC3 also plays an important role in the formation of hyperalgesia in the inflammatory environment. Inflammatory mediators can promote the transcription and expression level of the ASIC3, thus increasing the sensitivity of nociceptors [28].

### 3.3. Protease-Activated Receptor-2 (PAR-2)

PAR-2 is a G-protein-coupled receptor that also plays a key role in the pathogenesis of NERD. Activated by serine protease, PAR-2 can increase the secretion of IL-8 and contribute to the promotion of inflammation. Activated PAR-2 not only increases the release of the TRPV1 agonist but also participates in neurogenic inflammation at the same time. Activated PAR-2 and TRPV1 can induce the release of a neuropeptide, SP, which increases vascular permeability and activates leukocytes by binding to the neurokinin 1 receptor (NK1R), which plays an important role in pain transmission and neurogenic inflammation. Further, CGRP contributes to pain, inflammation, secretion, and movement through the heterodimer receptors composed of calcitonin receptor-like receptor (CRLR) and receptor activity modifying protein 1 (RAMP1) [13]. Activated PAR-2 can also mediate esophageal VH by enhancing the sensitivity of TRPV1 and ASICs. A study by Wu et al. [29] found that ATP released by human esophageal epithelial cells induced by weak acid reflux could activate PAR-2, which further led to the activation of TRPV1 and ASICs. In animal experiments, Suzuki et al. [30] found that PAR-2 activated by trypsin could further increase the release of ATP from mouse esophageal keratinocytes.

## 4. NERD and Abnormal Psychological Factors

A close relationship exists between the brain and the gastrointestinal tract, and many studies have explored the relationship between functional gastrointestinal diseases and psychosocial factors. For example, stress and emotion can affect gastrointestinal function and sometimes lead to gastrointestinal symptoms and diseases. In the same way, the state of gastrointestinal organs can affect one's emotional state. The symptoms of NERD often occur repeatedly, causing a heavy physical and mental burden on affected patients. These recurrent symptoms can cause long-term anxiety and depression or aggravate digestive-system-related symptoms [31]. Choi et al. [32] reported that the levels of anxiety and depression in patients with GERD—especially those with NERD—were significantly higher than those among the control group. Lei et al. [33] also reported that the risk of NERD in individuals with anxiety and depression was significantly higher than that in those without a history of mental illness.

### 4.1. NERD and Brain-Gut Axis

Mental and psychological factors may alter the sensitivity of the esophageal mucosa by affecting the brain-gut axis, thereby lowering the threshold of esophageal sensation and enhancing patients' responsiveness to low-intensity esophageal stimulation. The brain-gut axis is a two-way loop connecting the central nervous system and the gastrointestinal system; it primarily involves three nervous systems: the central nervous system (CNS), the autonomic nervous system (ANS), and the enteric nervous system (ENS). Any abnormal activity in these three systems can cause the abnormal regulation of the gastrointestinal tract. Among the three, the effects of the CNS on the gastrointestinal tract are mainly realized through the HPA axis. Broers et al. [34] found that the stress-activated HPA axis can directly or indirectly promote the release of gastrointestinal hormones, abnormally increase the sensitivity of the esophageal mucosa, and aggravate the symptoms of reflux. Moreover, CRH plays a key role in activating the HPA axis for endocrine regulation. Yamasaki et al. [35] demonstrated that esophageal sensitivity increases after the intravenous injection of CRH in a normal population, indirectly emphasizing the importance of psychological stress in esophageal VH. Brain-gut peptides, which can regulate visceral sensation, gastrointestinal motility, and mental psychology, are small molecular substances distributed within the brain and the intestines. A study by Desai et al. [36] demonstrated that an increased level of cholecystokinin (CCK) in brain-gut peptides increased anxiety and depression-like behavior and that this anxiety and depression led also to the abnormal secretion of brain-gut peptides (which is also one of the possible mechanisms of the vicious circle between emotional disorders and physical symptoms in patients with NERD). 5-Hydroxytryptamine (5-HT) is an important neurotransmitter in the cerebroenteric nervous system. Studies have shown that 5-HT plays an important role in the regulation of gastrointestinal sensation, movement, and sensitivity; the decrease of its secretion can lead to the relaxation of the esophageal smooth muscle and an increased sensitivity of the esophageal mucosa, as well as to the significant decrease in the blood level of 5-HT in patients with anxiety and depression [37, 38]. Further, sleep disturbance and depression promote each other [38]. A study by Lei et al. [33] found that the prevalence of NERD in patients with sleep disturbance issues was higher than that in patients in the control group. Total sleep deprivation can induce visceral hyperalgesia, potentially because sleep disturbances increase the activity of nitric oxide synthase (NOS) and its signaling pathways and affect the neural activity involved in the decrease in pain tolerance [40–42]. Sleep disturbances can also lead to the abnormal secretion of leptin, melatonin, and HPA axis hormones, which may also be mechanisms of VH caused by sleep disturbances [43–47].

### 4.2. NERD and Intestinal Microecology

In recent years, some scholars have found that intestinal microecological imbalances are closely related to mental and psychological abnormalities and VH. The intestinal tract contains the most complex microecosystem in the human body, and its diversity and stability are important for maintaining individual health. In recent studies, the composition of bacteria in fecal samples of patients with depression was found to be significantly different from that of those without depression. And the proportion of intestinal pathogenic Enterobacteriaceae and inflammatory bacteria in patients with depression was found to be significantly higher than that in those without depression [48]. At the same time, changes in the intestinal flora can induce depression: when the feces of patients with depression were transplanted into germ-free mice, depression-like behavior was induced [49]. Another study found that, in germ-free mice, the size of two pain-sensing areas changed: the anterior cingulate cortex became smaller, and the gray area around the midbrain aqueduct became larger [50]. Compared with normal mice, differences were found in the single nerve cells in the anterior cingulate cortex, characterized by VH and a decreased pain threshold. These forms of hypersensitivity can be reversed later in life, however, by microbes settling in the gut. The composition of the microbiota in patients with NERD is different from that of the control and RE patients, in that the NERD microbiota are composed of a higher level of Proteobacteria, Bacteroidetes, and Dorea spp. This increase in sulfate-reducing Proteobacteria spp. and Bacteroidetes spp., along with hydrogen-producing Dorea spp., may be involved in the presence of NERD VH [51]. Gram-negative bacterial products activate TLRs that, on the esophageal endothelial cells, cause an inflammatory cascade and a microbial-brain-gut axis, which may also be the mechanisms of NERD VH [52]. However, further experiments are needed to verify whether there is a direct correlation between the changes in the intestinal flora and the occurrence and development of NERD VH.

## 5. Discussion

**Figure 1:**
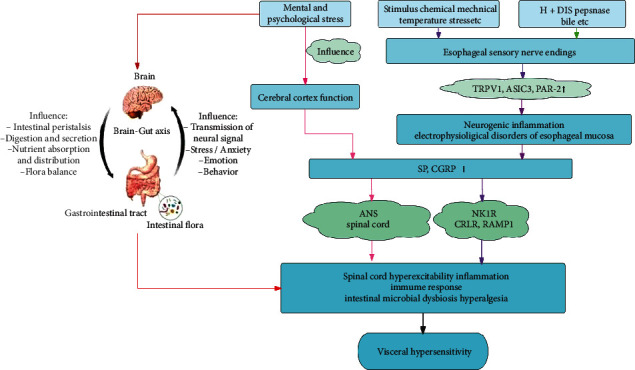
Endogenous or exogenous stimulation can activate acid-sensitive receptors, affect the function of the cerebral cortex, activate the brain-gut axis, and eventually lead to central hyperexcitability, inflammation, immune response, hyperalgesia, and intestinal microbial disorders through the release of neuropeptides (SP and CGRP) or brain-gut peptides. These factors jointly play an important role in the VH of NERD.

VH is one of the most important mechanisms of acid reflux, heartburn, and other related symptoms in patients with NERD. With recent research, scholars have gained a deeper understanding of the mechanism of VH in NERD. Peripheral and central nervous system sensitization mediated by neuropeptides SP and CGRP, the activation of acid-sensitive receptors, abnormalities of mental and psychological factors, and the brain-gut axis may all be involved in the occurrence of VH in NERD (Figure 1). Clarifying these mechanisms may provide new directions for the treatment of patients with NERD. In recent years, TRPV1 antagonists, tricyclic antidepressants, selective 5-HT reuptake inhibitors, 5-HT norepinephrine reuptake inhibitors, and other drugs have been widely used in the treatment of NERD, with good results. In addition, patient care and effective psychological intervention to relieve symptoms of anxiety and depression can help alleviate the symptoms of NERD. Studies have confirmed that the combination of psychological intervention and medication is more effective than medication alone in the treatment of NERD [53]. The generation of VH in NERD is a complex process with multiple factors. Current research remains in its initial stages, and the mechanisms have not been fully elucidated. The pathophysiological mechanism of VH in NERD requires a theoretical basis that can provide a new direction for developing a drug for NERD therapy.

## Figures and Tables

**Table 1:** NERD and acid-sensitive receptor expression level.

References	State	Research objects	Methods	Results
Kandulski et al. [16]	Germany	NERD group (*n* = 46) Healthy control group (*n* = 27)	Biopsies were taken at 2 cm above the squamocolumnar junction at the 3 o'clock position, PAR-2 expression was analyzed by quantitative reverse transcription- (RT-) PCR and immunohistochemistry.	PAR-2 protein expression: NERD group (6.57 ± 0.75) Healthy control group (2.3 ± 0.52) (*P* = 0.001)
Kim et al. [17]	South Korea	NERD group (*n* = 14) Healthy control group (*n* = 16)	Biopsies using standard biopsy forceps at a fixed position 3 cm above the squamocolumnar junction. PAR-2 RNA expression was assessed by qPCR.	PAR-2 mRNA expression: NERD group (2.39 ± 0.36) Healthy control group (1.37 ± 0.20) (*P* = 0.020)
Yoshida et al. [13]	Japan	NERD group (*n* = 24) Healthy control group (*n* = 24)	Biopsies for mRNA analysis were taken from the esophageal squamous mucosa at 5 mm above the squamocolumnar junction. The mRNA expression level of TRPV1 and PAR-2 was assessed by real-time RT-PCR and enzyme immunoassay.	Compared with the healthy control group, mRNA of TRPV1 and PAR-2 were significantly elevated in the NERD group (*P* < 0.05). TRPV1 mRNA in the healthy control group could not be detected
Guarino et al. [18]	Italy	NERD group (*n* = 9) Healthy control group (*n* = 7)	Biopsies were taken from the esophageal squamous mucosa at 5 cm above the squamocolumnar junction, relative optical density of TRPV1 protein was assessed by Western blot analysis, and TRPV1 RNA expression was assessed by qPCR.	TRPV1 mRNA expression: NERD group (1.98 ± 0.21) Healthy control group (1.00 ± 0.06) (*P* < 0.01) Relative optical density of TRPV1 protein: NERD group (0.65 ± 0.07) Healthy control group (0.34 ± 0.04) (*P* < 0.01)
Ustaoglu et al. [19]	Britain	NERD group (*n* = 10) ERD group (*n* = 10) BE group (*n* = 8)	Biopsies obtained from the distal esophageal mucosa were costained with TRPV1; TRPV1 RNA expression was assessed by qPCR.	NERD patients had significantly increased expression of TRPV1 on superficial sensory nerves compared to ERD (*P* = 0.028) or BE (*P* = 0.017)
Silva et al. [20]	Brazil	Experimental group (NERD was surgically induced in Swiss mice) Control group (sham surgery)	Collect mice esophagus; TRPV1 protein expression was assessed by Western blot analysis.	TRPV1 protein expression: Experimental group (3.33 ± 0.66) Control group (1.00 ± 0.07) (*P* < 0.05)
Patcharatrakul et al. [21]	Thailand	NERD group (*n* = 15) Healthy control group (*n* = 15)	Two groups underwent single-photon emission computed tomography after ingesting 2 g of chili or placebo in capsules in a randomized double-blind crossover fashion with a one-week washout period to compare gastric accommodation (GA).	GA: NERD group (451 ± 89 mL) Healthy control group (375 ± 81 mL) (*P* < 0.05)

RT-PCR: reverse transcription-polymerase chain reaction; qPCR: quantitative real-time polymerase chain reaction; ERD: erosive reflux disease; BE, Barrett esophagus; GA: gastric accommodation.
